# Surgery for Hypertrophic Obstructive Cardiomyopathy: Comprehensive LVOT Management beyond Septal Myectomy

**DOI:** 10.3390/jcm10194397

**Published:** 2021-09-26

**Authors:** Alessandro Affronti, Robert Pruna-Guillen, Elena Sandoval, Daniel Pereda, Jorge Alcocer, Manuel Castellà, Eduard Quintana

**Affiliations:** Cardiovascular Surgery, Institut Clínic Cardiovascular, Hospital Clínic de Barcelona, University of Barcelona Medical School, c/Villarroel 170 Esc 1 5th Floor, 08036 Barcelona, Spain; affronti@clinic.cat (A.A.); pruna@clinic.cat (R.P.-G.); esandova@clinic.cat (E.S.); dpereda@clinic.cat (D.P.); alcocer@clinic.cat (J.A.); mcaste@clinic.cat (M.C.)

**Keywords:** hypertrophic obstructive cardiomyopathy, systolic anterior motion, septal myectomy, mitral valve

## Abstract

Hypertrophic cardiomyopathy (HCM) is a complex, underestimated, multifaceted disease frequently associated with left ventricular outflow tract (LVOT) obstruction. It is clearly demonstrated that this is due not only to septal hypertrophy but also to systolic anterior motion (SAM) of mitral valve leaflets secondary to mitral valve/subvalvular apparatus abnormalities. Surgical treatment involves performing an extended septal myectomy, eventually followed by ancillary procedures to those structures responsible for maintaining LVOT obstruction, if necessary. In this review, we describe the spectrum of possible surgical techniques beyond septal myectomy and their pathophysiologic rationale.

## 1. Introduction

Hypertrophic cardiomyopathy (HCM) is defined as a disease characterized predominantly by left ventricular hypertrophy in the absence of another cardiac, systemic, or metabolic disease, explaining the hypertrophic state. A ≥15 mm myocardial wall thickness anywhere in the left ventricle is considered the cut-off value to establish diagnosis. The typical finding in HCM variants is an asymmetric septal hypertrophy pattern with more or less involvement of the septum from the base to the apex of the left ventricle. However, many different phenotypes exist regardless of the presence of obstruction or not (apical, concentric, lateral wall, and right ventricular) [1].

Hypertrophic cardiomyopathy (HCM) is associated with an autosomal dominant transmission of alterations in genes encoding proteins of the cardiac sarcomere or sarcomere-related structures. The exact way the genetic alteration produces a dysfunctional histological structure with myocyte disarrangement, fibrosis, and hypertrophy has not been elucidated in detail [2,3].

On the other hand, for a considerable percentage of patients with HCM, it is not possible to identify any associated genetic alteration, nor the involvement of other members of the family (the so called “non-familial” HCM) [4].

Thus, it is likely that further pathophysiologic mechanisms may be responsible for the clinical expression of the disease.

## 2. Prevalence: The Real Burden of the Disease in 2021

Historically, the prevalence of HCM has been reported at about 1:500. This figure was originally extrapolated from the echocardiographic findings of a cohort of young participants of the CARDIA (Coronary Artery Risk Development in Young Adults) study [5]. However, advances in different fields related to HCM in recent years made previous studies epidemiologically outdated, and it is likely that the burden of the disease may have been underestimated. Semsarian and coworkers, considering the clinically expressed HCM and the gene carriers, estimated the actual prevalence at about 1:200 [6].

## 3. Main Features: Left Ventricular Outflow Tract (LVOT) Obstruction, Systolic Anterior Motion (SAM), and Mitral Regurgitation

The left ventricular outflow tract (LVOT) obstruction is considered the hallmark of HCM, even though it is only present in approximately 75% of cases [7].

Advances in cardiovascular imaging, especially echocardiography and cardiac magnetic resonance (CMR), in the last 15–20 years, as well as direct observation during surgery, allowed for a better understanding of the mechanisms underlying LVOT obstruction in HCM patients. While initially septal hypertrophy alone was thought to be the only one responsible [8,9], currently it is evident that LVOT obstruction is a dynamic process where alterations of the mitral valve or the subvalvular apparatus play an important role in the majority of patients [10,11,12]. These structural alterations may determine direct obstruction of LVOT or, more frequently, promote systolic anterior motion (SAM).

According to the classical theory, SAM would be secondary to the septal hypertrophy that causes narrowing of the LVOT. The high velocities developed at this level because of the Venturi effect would cause a suction of the mitral valve leaflets towards the septum [13].

**Figure 1:**
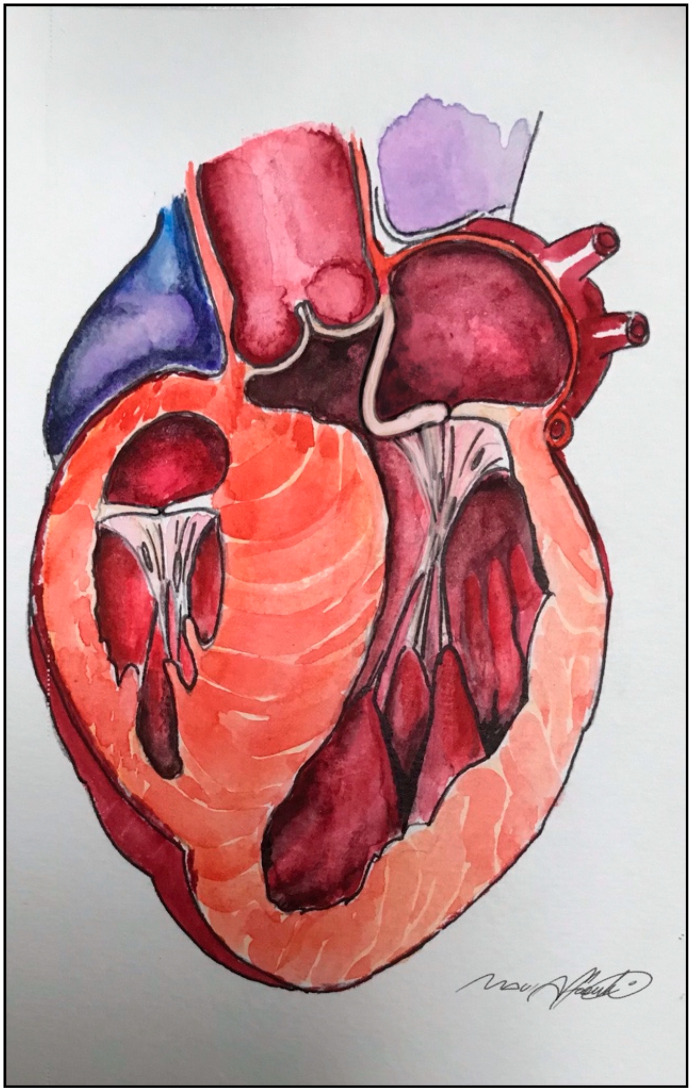
An elongated anterior mitral leaflet contributing to secondary systolic anterior motion (SAM) and left ventricular outflow tract (LVOT) obstruction.

A more contemporary theory, based on echocardiographic observations, presupposes the role of the direct force of ejection flow during early systole, striking the ventricular aspect of mitral leaflets and dragging them towards the septum [14,15]. Once the leaflets and the septum are in contact, it is the gradient of pressure itself that maintains the obstruction [16], Figure 1 and Video S1.

**Figure 2:**
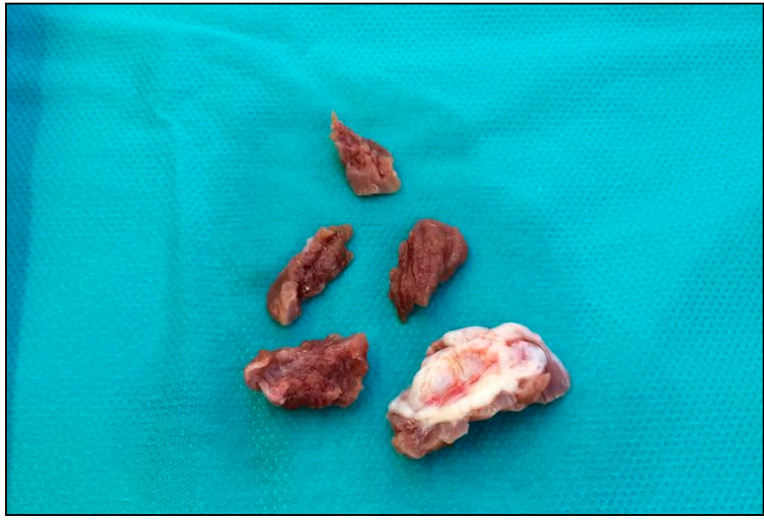
Surgical specimen showing the typical endocardial fibrous plaque (contact lesion) produced by the repeated contact of the mitral leaflets with the hypertrophic septum.

The repeated contact of the valve leaflets with the septum is macroscopically evident as an endocardial fibrous plaque (contact lesion) [17], Figure 2.

The hypertrophic septum also contributes to the process, redirecting the ejection flow posteriorly and laterally in the left ventricular cavity, towards the mitral valve [18].

In summary, this overlap of the inflow and outflow portions of the left ventricle (LV), which is crucial in determining SAM, presupposes three identifiable conditions: the anterior positioning of the mitral leaflet in the LV cavity, a certain degree of chordal slack that determines an increased mobility of the valve, and third, LV ejection stream dragging the mitral leaflets into the septum [18,19].

The first two conditions may be secondary to different combinations of structural abnormalities of the mitral valve and its apparatus that are frequently associated with HCM.

When SAM occurs, there is an asymmetry and reduction in leaflets’ coaptation surface, which translates into a variable degree of mitral regurgitation (MR). The resulting jet is typically posterior or laterally oriented [20].

Structural alterations of the mitral valve may also concur in determining MR.

## 4. Indication for Surgery

The clinical expression of HCM may vary extremely. It usually presents symptoms of dyspnea, angina, and/or syncope, and it may be associated with sudden death and heart failure. The initial treatment for symptomatic patients with obstruction is medical therapy with β -blockers, calcium antagonists, and disopyramide. In case of the persistency of symptoms, despite optimal medical therapy, a septal reduction therapy, either surgical myectomy or in some cases alcohol septal ablation (ASA), is necessary; indeed, it has been demonstrated that in such patients, LVOT obstruction at rest is a strong, independent predictor of progression to severe symptoms of heart failure and death [21].

Transaortic extended septal myectomy is currently considered the most appropriate treatment for the majority of severely symptomatic patients with obstructive HCM, when unresponsive to medical therapy.

In fact, according to the recent 2020 ACC/AHA guidelines:

“In patients with obstructive HCM who remain severely symptomatic despite guideline-directed medical therapy, septal reduction therapy (SRT) in eligible patients, performed at experienced centers, is recommended for relieving LVOT obstruction”

(COR I, LOE B-NR).

More specifically, “In symptomatic patients with obstructive HCM who have associated cardiac disease requiring surgical treatment (i.e., associated anomalous papillary muscle, markedly elongated anterior mitral leaflet, intrinsic mitral valve disease, multivessel CAD, valvular aortic stenosis), surgical myectomy, performed at experienced centers, is recommended” (COR I, LOE B-NR) [1].

It is worth noting that in the previous edition of the American guidelines, surgery was recommended as COR II, LOE B. The upgrade to a class I recommendation is an important recognition of its essential role over alcohol septal ablation (ASA) as first line treatment for the majority of patients requiring an invasive treatment.

The European Society of Cardiology (ESC) 2014 guidelines recommend septal reduction therapy for patients with a maximum resting or provoked LVOT gradient of ≥50 mm Hg, who are in NYHA functional Class III–IV, despite maximum tolerated medical therapy (COR I, LOE B).

Again, “Septal myectomy, rather than ASA, is recommended in patients with an indication for septal reduction therapy and other lesions requiring surgical intervention (e.g., mitral valve repair/replacement, papillary muscle intervention)”. (COR I, LOE C) [22].

## 5. Extended Septal Myectomy: Evolution of a Technique

After the early anecdotic descriptions from various surgeons in the US and Europe in the 1950s, the first systematic attempts to develop an effective surgical treatment for HCM were those of Kirklin and, subsequently, Frye, both from the Mayo Clinic [23,24].

Almost at the same time, Morrow proposed his technique, which rapidly gained popularity in the surgical community.

The procedure consisted in creating a rectangular channel in the interventricular septum. According to the author, the depth was more important than the length (the extension) of the incision [25].

In 1994, for the first time, Messmer and colleagues introduced the concept of extended myectomy. The technique was based on the use of a sharp triple-hook retractor positioned underneath the deepest part of the septal ridge. A cephalad traction on it allowed distal septum removal.

Excision was then extended beyond the distal septum towards the LV wall, and any secondary anomalous attachment with the LV wall and papillary muscles was also resected. Moreover, for the first time the authors pointed out the importance of a systematic inspection of the mitral apparatus as an integral part of the procedure [26].

The current extended myectomy technique was introduced by the Mayo Clinic group [17]. A preprocedural intraoperative TEE is routinely performed in order to confirm the surgical plan or add new information.

An oblique or transverse aortotomy is performed slightly lower than that used for aortic valve replacement. After identifying the fibrotic area in the septum (the contact lesion), expression of the mitral-septal interaction because of the SAM, the incision starts just at of the nadir of the right aortic cusp, continuing upward and leftward towards the left trigone. It is important at this stage to leave enough tissue underneath the right cusp to avoid prolapse and subsequent regurgitation (usually at least 0.5 cm). After the first resection, the incision is then further deepened and extended beyond the endocardial scar towards the apex. A sponge stick pushing the anterior wall may improve the distal septum exposure. Any trabeculation or abnormal chordae are divided. Ideally, the depth of the resected part should be two-thirds the thickness of the septum, and the residual septum should be at least 1 cm thick.

As opposed to the Morrow technique, in this case the core concept is that the extension is more important than the depth. According to the authors, in fact, the persistency of a residual gradient and SAM is frequently due to a septal myectomy not sufficiently extended towards the apex [17].

## 6. Left Ventricular Outflow Tract Management beyond Septal Myectomy

It has become evident that a significant portion of patients with obstructive HCM present a certain degree of mitral valve apparatus abnormalities that predispose to SAM and LVOT obstruction [27,28].

This is particularly irrefutable in patients presenting with dynamic LVOT obstruction in the absence of a significant basal septal hypertrophy.

Indeed, mitral valve structural alterations have been described since the beginning of studies on HCM [29,30,31,32]; however, for many years they have not been considered important from the pathophysiologic point of view, and therapeutic intervention limited to the septal myocardium.

At most, if necessary, a mitral valve replacement (MVR) was recommended [33].

In recent years, a clearer awareness of the importance of mitral structural abnormalities in obstructive HCM has led to a broader use of specific techniques to address this aspect of LVOT obstruction.

Currently, MVR is reserved for patients with intrinsic leaflet abnormalities that cannot be repaired [17] or calcification of the leaflets or the annulus that would not allow a mitral-sparing strategy [34].

As commented by Minakata et al., regarding MVR in this setting, “the major disadvantage of this procedure is that one disease process is replaced with others such as problems of durability, infection, thromboembolism and anticoagulation, which are associated with prosthetic valves” [35].

In this light, a thorough preoperative and intraoperative study should include transthoracic (TTE) and transesophageal echocardiography (TEE), as well as cardiac magnetic resonance (CMR) if necessary.

A wide variety of mitral valve structural abnormalities have been identified at all levels in HCM patients. It is important to note that none of the concomitant LVOT interventions described here increases the risk of iatrogenic atrio-ventricular block; thus, the risk of requiring a permanent pacemaker after surgery remains in the range of 2–3% [36].

## 7. Elongation of Mitral Leaflets

Elongation of mitral leaflets is probably the most frequent anomaly associated with obstructive HCM. Klues and associates found that 44% of patients with obstructive HCM had enlarged and elongated mitral valves leaflets [12].

Jiang and coworkers demonstrated that mitral leaflets of patients with obstructive HCM and SAM were on average 1.5–1.7 mm longer than those of patients with HCM but no SAM, and those from normal subjects [37].

Elongated leaflets protrude in the LV ventricle and tend to coapt at a lower level than the normal valvular plane predisposing to SAM and LVOT obstruction [29]. The resulting typical echocardiographic image has been called “the nightcap” [34].

Different surgical techniques have been advocated to treat anterior mitral leaflet (AML) elongation.

The vertical plication, proposed by McIntosh et al., consists of a plication of the AML along its long axis to reduce the billowing; however, such a technique could alter the coaptation line, resulting in central MR [38].

**Figure 3:**
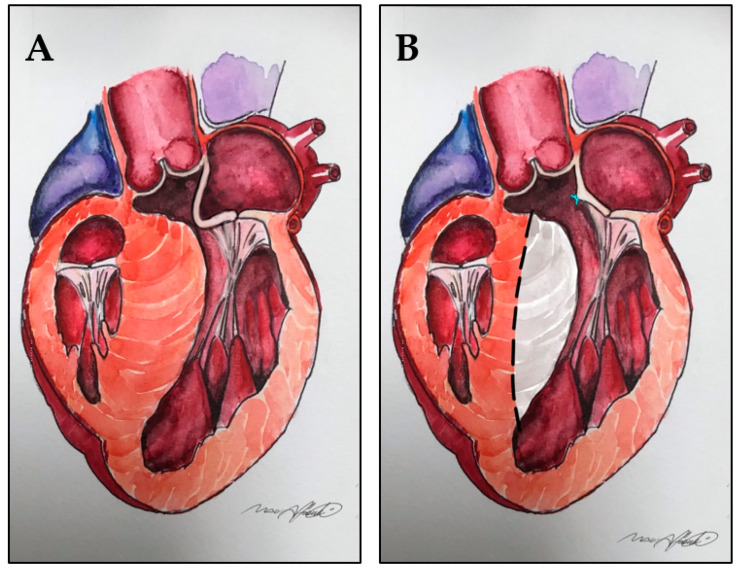
(**A**): elongated anterior mitral leaflet contributing to LVOT obstruction. (**B**): anterior mitral leaflet plication reduces the surface exposed to the LVOT without altering the coaptation line. Dotted line and gray area: extended septal myectomy.

**Figure 4:**
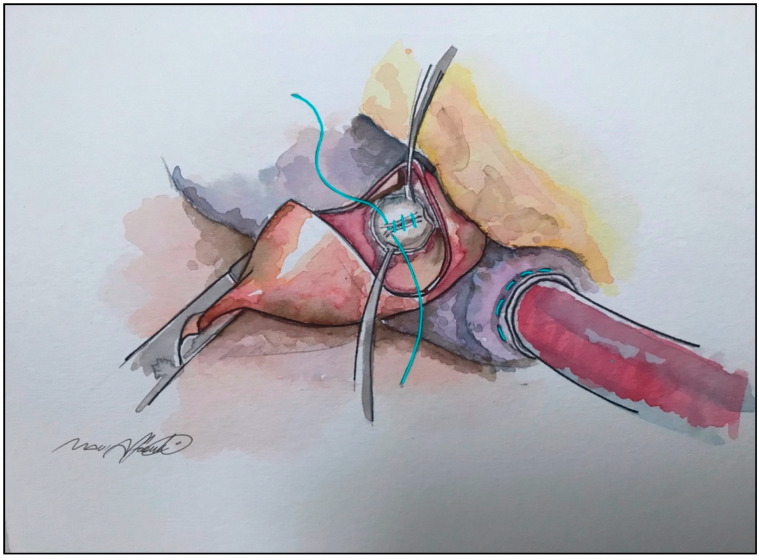
Anterior mitral leaflet plication from transaortic approach.

More effective and reproducible seems to be the horizontal plication, which is part of the so-called RPR procedure: resection–plication–release, described by Swistel and coworkers. It consists in a series of 5–0 fine vertical mattress single stitches along the short axis of AML, in the main body of the leaflet, just below the mitro-aortic curtain [39]. Figure 3 and Figure 4.

This technique is performed through the same transaortic approach of the myectomy, and it allows a stiffening and shortening of the leaflet without altering the coaptation line, resulting in a reduced surface exposed to the LVOT. Usually, it permits a reduction between 2 and 5 mm, depending on the extent of correction needed, evaluated preoperatively by echocardiography and confirmed by direct vision of the slack and redundancy of AML [39,40]. Apart from its simplicity and fast execution, another advantage is that it can be easily removed or adjusted in case of MR in intraoperative, post-procedural echocardiography.

Another technique is the anterior mitral leaflet extension (AMLE), which involves the vertical incision of the AML, followed by the suture of an oval autologous pericardial patch pre-treated with Glutaraldehyde.

The idea is to stiffen the central portion of the AML, reducing its abnormal mobility, which favors SAM. Furthermore, the patch extends the width of AML but not its length, determining a lateral shift of the central-attached chordae tendineae that result more under tension. This reduction in chordal slack also contributes to the reduction of SAM [41,42].

A possible criticism of this technique is that it may be time consuming compared to other surgical options, adding extra time to cross-clamp and cardiopulmonary bypass (CPB) duration. Calcification of the pericardial patch is another possible drawback; however, the same working group published the long-term outcomes of 98 patients treated with this approach, along with an extended myectomy, and after a follow-up of 8.3 ± 6.1 years, 88% of patients were free of reoperation [43].

Sometimes, the tip of an elongated AML may extend beyond the coaptation line, further contributing to SAM [44].

This residual portion of the leaflet, usually at A2 level, does not contribute to coaptation, and it is usually associated with a bunch of extremely slack and thinned chordae tendineae. In such cases, this segment can be plicated or easily removed without determining flail, Video S2.

## 8. Displacement of the Anterolateral Papillary Muscle and Abnormal Connections with Left Ventricle Free Wall

In patients with obstructive HCM, the base of the anterolateral papillary muscle may be anteriorly displaced, determining an apical shift of the mitral valve leaflets [45,46]. The anterolateral papillary muscle may also present abnormal connections with the LV anterolateral wall [40].

Both conditions may favor SAM and LVOT obstruction since, due to leaflet elongation, the coaptation area lies in the LV cavity, exposed to ejection flow.

The modern techniques of extended myectomy may address this problem, freeing the papillary muscle from all the secondary attachments, as well as cutting it down from the ventricular wall or trim it, if necessary [17,26].

Restoring a more physiological position of the papillary muscle allows a separation of the inflow and outflow portions of the LV, thus preventing the ejection flow from dragging the mitral leaflets towards the LVOT [16].

## 9. Bifid and Hypermobile Papillary Muscles

Kwon et al. demonstrated that patients with dynamic LVOT obstruction may present bifid and hypermobile papillary muscles.

When this occurs, the papillary muscles are at risk of being dragged into the LVOT, determining dynamic obstruction, even in the absence of a significant septal hypertrophy [47].

**Figure 5:**
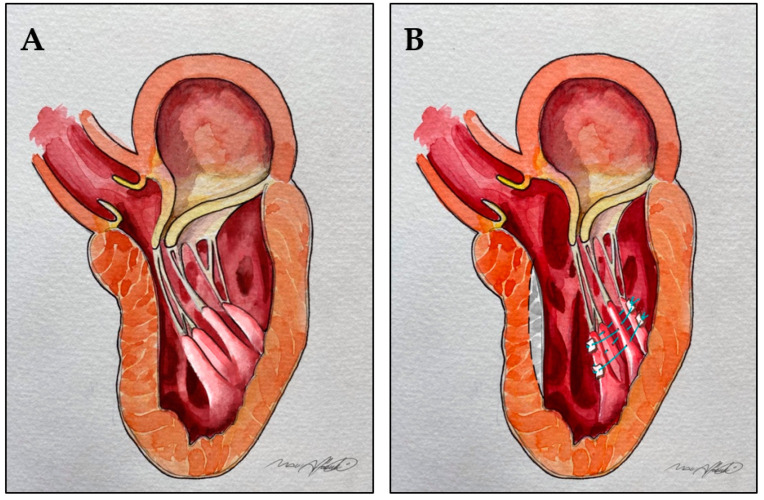
Reorientation of hypermobile bifid papillary muscles. (**A**): Bifid hypermobile papillary muscles determining LVOT obstruction. (**B**): Pledgetted mattress sutures are used to reposition the mobile papillary muscles towards the posterior wall of the left ventricle, thus pulling them away from the LVOT. Dotted line and gray area: shallow septal myectomy.

Surgical reorientation involves the use of pledgeted mattress sutures, placed posterior to the most posterior papillary muscle head and then passed through the anterior one. The sutures are then tied to fix the mobile papillary muscle towards the posterior left ventricle, to reduce its excessive mobility, and pull it away from the LVOT [48], Figure 5.

## 10. Insertion of the Anterolateral Papillary Muscle Directly into the Anterior Mitral Leaflet

Another described feature in patients with obstructive HCM is the direct insertion of a head of the anterolateral papillary muscle into the midportion of the AML, without interposition of chordae tendinae [49].

In addition, there is often the concomitant fusion of the papillary muscle to the left ventricular septum or free wall [17].

This can cause direct obstruction of the LVOT, especially when the papillary muscle is particularly thick. The abnormal muscle can be easily removed, providing that it does not support the free edge of the leaflet. If this is not possible because of the risk of flail, the resection can be limited to the anterior portion of the muscle, leaving the posterior part attached. Finally, in the case of a papillary muscle that is especially thick, it can be shaved to its base to make it thinner [35].

## 11. Anterior Mitral Leaflet Tethering by Secondary Order Chordae

While normally secondary order chordae are important in maintaining LV shape and function during contraction, in HCM patients they frequently appear fibrotic and/or retracted and determining tethering of the AML toward the apex and LVOT [28]. The identification and selective cut of these chordae allows the repositioning of the coaptation point more physiologically, farther from the outflow tract. The approach is conceptually similar to the cutting of strut chordae to treat functional mitral regurgitation [50], Video S3.

Ferrazzi et al. reported a series of 39 symptomatic patients with severe LVOT obstruction, but moderate septal hypertrophy (≤19 mm), treated by chordal cutting in association with a shallow septal myectomy. When compared with the control group (similar characteristics but treated with a shallow myectomy only), the authors found a 27% post-operative increase in the AML–annulus ratio, which expresses a more physiological leaflets coaptation point, farther from the LVOT. In the control group, this increase was only 3%.

Another interesting result in the chordal cutting cohort is a 29% post-operative decrease of the MV tenting area, which suggests a reduction of leaflets displacement towards the apex. The post-operative tenting area did not change significantly in the control group.

All the patients treated with chordal cutting improved clinically (NYHA I and II) and echocardiographically, in terms of gradient [51].

## 12. Mitro-Aortic Discontinuity

A recent study from the same working group demonstrated another possible alteration of the mitral valve associated with HCM: the so-called muscular mitral-aortic discontinuity. Normally, the AML is in direct continuation with the aortic valve, particularly with the left and non-coronary leaflets, to form the mitro-aortic continuity (also known as the mitro-aortic curtain).

The presence of a muscular band between the two valves has been previously documented in patients with abnormal ventriculoarterial connections, such as the double outlet right ventricle [52,53].

In their study, the authors found this anatomic feature in 28 of 106 young patients undergoing surgery for HCM (26%). Interestingly, patients with the anomaly were significantly younger than those without. The genetic analysis revealed that sarcomere gene mutations were present in 22 of the 28 patients (79%) with muscular discontinuity, versus only 24 (36%) in the negative group (*p* > 0.001).

Patients with mitro-aortic discontinuity showed a significantly longer AML on echocardiogram compared to those with a normal mitro-aortic continuity.

Thus, the presence of the muscular band could predispose to AML elongation or be a consequence of embryologic derangements associated with mitral abnormalities, such as elongated AML [54].

## 13. “Edge-to-Edge” Technique

Occasionally, mitral valve abnormalities in the setting of obstructive HCM have been treated with the so called “edge-to-edge” technique [55].

Shah and coworkers presented a small series of 24 patient with obstructive HCM associated with SAM due to an elongated AML and moderate or greater mitral regurgitation. They report positive clinical and echocardiographic results; however, this approach is not always feasible, especially because of the difficulty in adequately exposing the posterior leaflet [56] resulting in a potentially increased risk of mitral stenosis, especially in patients with a small annulus [57,58]. Thus, the technique in this setting should probably be used only as a bailout option in selected cases to avoid MV replacement.

## 14. Outcomes: The Importance of a Tailored Approach and Dedicated High Expertise Referral Centers

As in the case of valvular or coronary disease, where the institution of the Heart-team has improved the discussion and refined the decision-making process, patients with HCM should be assessed and followed by a multidisciplinary team, in a referral center. In fact, because of the intrinsic variability and complexity of the disease, a dedicated team ensures the patient receives a comprehensive evaluation and a tailored intervention. With improvements in echocardiographic assessment of LV geometry and mitral-septal interaction with use of real time 3D echo, better preoperative and intraoperative understanding of complex pathophysiologies may translate into more predictable intervention and outcomes [59].

This aspect is clearly supported by the current 2020 American guidelines which recommend that the procedure should be performed at experienced centers (comprehensive or primary HCM centers) with demonstrated excellence in clinical outcomes for these procedures (COR I, LOE C-LD) [1].

Similarly, the 2014 ESC guidelines advocate that septal reduction therapies are performed by experienced operators, working as part of a multidisciplinary team of experts in the management of HCM (COR I, LOE C) [22].

Surgical myectomy for obstructive HCM, indeed, is today one of the safest cardiac surgical interventions, when performed in high-volume, high-expertise centers.

Maron et al. analyzed the last 15 years of such procedures, performed at the five main high-volume North American HCM institutions (Mayo Clinic, Cleveland Clinic, Toronto General, Tufts Center in Boston, and Mount Sinai/St. Luke/Roosevelt in New York). All of them shared similar excellent outcomes, and the composite 30-day mortality rate of the five institutions was 0.4% [60], which is lower than the mortality rate of the majority of standard cardiac surgical procedures, such as coronary artery bypass grafting (2.3%), aortic valve replacement (3.4%), mitral valve replacement (5.7%), and mitral valve repair (1.6%), according to statistics from the Society for Thoracic Surgery (STS) [61].

Excellent results are also achieved in the relief of symptoms (90–95%) [62,63] and survival free from sudden death [64]. Furthermore, long-term survival is similar to that of an age-matched general population [65,66].

The above-mentioned mortality rate is in stark contrast with the 5.9% published by Panaich and colleagues, who extrapolated data from the Healthcare Cost and Utilization Project’s Nationwide Inpatient Sample. The authors also reported an 8.7% rate of complete heart block. These data depict the reality of the US’s community and low-volume hospitals: “the real word” outside the biggest HCM centers [67].

Kim et al., in 2016, provided a further insight on the situation across the US, analyzing the US nationwide inpatient database.

Surprisingly, the median number of septal reduction cases (either surgical myectomy or alcohol ablation) in this database is ≤1 per year, with a certain discrepancy amongst centers. Institutions and relative results were divided into three terciles, according to procedure volumes. The in-hospital mortality rate after surgical septal myectomy was an unacceptable 15.6% in the low-volume hospitals tercile but still 3.8% in the high-volume hospitals tercile, which is still approximately 10 times higher than that observed in the HCM referral centers [68].

This wide discrepancy in mortality corroborates the importance of centralizing HCM patients in dedicated, high-volume referral centers with highly experienced professionals.

In this setting, as advocated by Maron and colleagues, it would be worth promoting the creation of more HCM centers, considering also that the actual prevalence of the disease is likely much higher than what was believed in the past [5].

Similar planning would provide people easier access to the best treatment options for this complex disease [60].

## 15. Conclusions

A significant number of patients with obstructive HCM present mitral valve/subvalvular apparatus abnormalities contributing to LVOT obstruction.

In this setting, it is important to elucidate the specific mechanism underlying obstruction in order to offer a tailored and effective treatment to each patient. Ancillary procedures beyond extended septal myectomy should be part of the surgical armamentarium of any contemporary myectomy surgeon.

Ideally, surgery should be performed in high-expertise, high-volume centers.

## Data Availability

Not applicable.

## Supplementary Materials

The following are available online at https://www.mdpi.com/article/10.3390/jcm10194397/s1, Video S1. TEE, mid-esophageal long axis view showing elongated anterior mitral leaflets protruding in the LV ventricle determining SAM, LVOT obstruction, and mitral regurgitation. Video S2. Pre-operative TEE, mid-esophageal long axis view showing the tip of an elongated anterior mitral leaflet extending beyond the coaptation line and contributing to LVOT ostruction. Plicature of the free margin (A2) of the anterior leaflet. Post-operative TEE. Video S3. Chordal cutting technique: fibrotic and thickened secondary chordae are resected to relieve tethering of the anterior mitral leaflet.
