# Supplementary material for: Surgery for Hypertrophic Obstructive Cardiomyopathy: Comprehensive LVOT Management beyond Septal Myectomy
Source: J Clin Med. 2021 Sep 26;10(19):4397. doi: 10.3390/jcm10194397 (PMC8509570; doi:10.3390/jcm10194397)
Supplement: Supplementary file 1 [file jcm-10-04397-s001.zip › video legend.pdf]

Video S1. TEE, mid-esophageal long axis view showing elongated anterior mitral leaflets protruding in the left ventricle determining SAM, LVOT obstruction and mitral regurgitation.

Video S2. Pre-operative TEE, mid-esophageal long axis view showing the tip of an elongated anterior mitral leaflet extending beyond the coaptation line and contributing to LVOT ostruction. Plicature of the free margin (A2) of the anterior leaflet. Post-operative TEE.

Video S3. Chordal cutting technique: fibrotic and thickened secondary chordae are resected to relieve tethering of the anterior mitral leaflet.
